# The Role of Histone Modification in DNA Replication-Coupled Nucleosome Assembly and Cancer

**DOI:** 10.3390/ijms24054939

**Published:** 2023-03-03

**Authors:** Yaguang Zhang, Qin Zhang, Yang Zhang, Junhong Han

**Affiliations:** State Key Laboratory of Biotherapy and Cancer Center, and Frontiers Science Center for Disease-Related Molecular Network, West China Hospital, Sichuan University, Chengdu 610041, China

**Keywords:** histone modification, nucleosome assembly, DNA damage repair, cancer, epigenetics

## Abstract

Histone modification regulates replication-coupled nucleosome assembly, DNA damage repair, and gene transcription. Changes or mutations in factors involved in nucleosome assembly are closely related to the development and pathogenesis of cancer and other human diseases and are essential for maintaining genomic stability and epigenetic information transmission. In this review, we discuss the role of different types of histone posttranslational modifications in DNA replication-coupled nucleosome assembly and disease. In recent years, histone modification has been found to affect the deposition of newly synthesized histones and the repair of DNA damage, further affecting the assembly process of DNA replication-coupled nucleosomes. We summarize the role of histone modification in the nucleosome assembly process. At the same time, we review the mechanism of histone modification in cancer development and briefly describe the application of histone modification small molecule inhibitors in cancer therapy.

## 1. Introduction

The nucleosome is the basic unit of chromatin. It is an octamer composed of 4 core histones (H3, H4, H2A, H2B), including one H3-H4 tetramer and two H2A-H2B dimers, surrounded by 147 pairs of DNA base pairs [1]. The core histones form a spherical core particle, and their N-terminal tails are free from the core particle, which helps the modification occur. Posttranslational modifications (PTMs) are involved in a variety of cellular processes, such as transcription, DNA damage, apoptosis, and cell cycle regulation. Mass spectrometry is a powerful tool for finding and verifying histone PTMs [2]. In addition, new proteomic, genomic, and functional solid-phase chemistry tools have been developed to detect the function of PTMs [3,4].

## 2. Posttranslational Modifications of Histones

**Figure 1:**
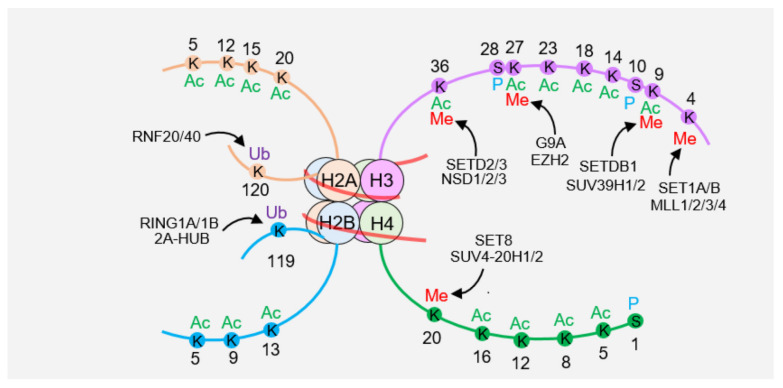
Schematic diagram of histone modification landscape. The nucleosome consists of one H3-H4 tetramer, two H2A-H2B dimers, and the surrounding DNA. There are many modifications on the histone tail, including histone methylation (Me), acetylation (Ac), ubiquitination (Ub), and phosphorylation (P).

Histone acetylation was first identified by biologist Vincent Allfrey in the 1960s and has been associated with mammalian gene activity [5,6]. Since then, histone PTMs have been discovered and well described. Currently, more than 10 different covalent modifications have been found on different amino acid residues of core histones, including acetylation of lysine, methylation of arginine and lysine, ubiquitination, ADP ribosylation, citrullination, phosphorylation of serine and tyrosine, isomerization of proline, sumoylation, carbonylation, and controversial biotinylation. Moreover, new modification sites and patterns are continuously discovered (Figure 1). Covalent modification can occur not only in the N-terminal tail protruding from the nucleosome but also in the core region. Different combinations of modification sites and forms can encode very rich information, which can be transmitted to daughter cells as epigenetic markers [7]. Therefore, the “histone code” hypothesis has been proposed by Allis et al. in the early 21st century and supposes that the functions derived from rich histone language are extremely extensive and fine, involving all aspects of cell fate determination, such as replication of genetic code, cell adaptation to internal and external environmental changes, regulation of gene expression, and others. Interpreting these messages and elucidating their functions is the main content of epigenetics [8,9,10].

Common sites of histone acetylation are K5, K9, and K13 on histone H2A [11,12]; K5, K12, K15, and K20 on H2B [13]; K9, K14, K18, K23, K27, K56, and K79 on H3 [11]; and K5, K8, K12, K16, and K91 on H4 [14]. The various acetylation sites of histones correspond to different functions. For example, H3K56 acetylation is involved in the regulation of nucleosome assembly, while H4K16 acetylation is involved in the regulation of nucleosome-mediated chromatin compaction, activation or inhibition of gene transcription, DNA damage repair, and other processes. Methylation occurs mainly at K4, K9, K27, K36, and K79 on histone H3 and at K20 of H4 [12]. Most histones are monoubiquitinated rather than polyubiquitinated, which occurs mainly on H2A and H2B [15,16].

## 3. Nucleosome Assembly

**Figure 2:**
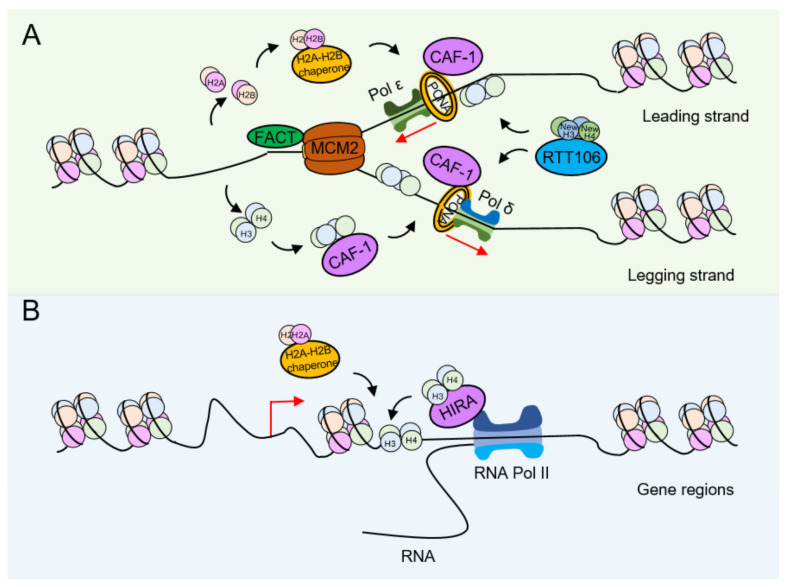
Diagram of RCNA and RINA. (**A**) Replication-coupled nucleosome assembly (RCNA). Nucleosomes must be disassembled to make way for DNA replication machinery. Nucleosomes are then reassembled in close concert with DNA replication on the leading and lagging strands. (**B**) Replication-independent nucleosome assembly (RINA). Many of the same core principles of nucleosome disassembly, DNA access and nucleosome assembly are likely applicable to replication-independent processes such as gene transcription.

A key feature of chromatin assembly during DNA replication is that it occurs immediately after DNA synthesis, with the first deposited nucleosome detected approximately 250 bp behind the replication fork. Histone deposition and chromatin assembly are important processes throughout S phase DNA synthesis and are indispensable for gene expression [17]. Histone chaperones, including Nap1, Vps75, NASP, FACT, CAF-1, Rtt106, Spt6, Asf1, and DAXX, play a central role in both histone deposition and chromatin assembly and are therefore involved in the regulation of cellular processes [18]. Nucleosomes can block DNA access during the S phase of the cell cycle [19]. Nucleosomes located in front of replication forks are depolymerized so that DNA replication elements can bind to DNA. During DNA replication, parental histones undergo depolymerization before replication forks, and newly synthesized histones are deposited on DNA with the help of histone chaperones to reform nucleosomes and assemble chromatin [20]. Newly synthesized and preexisting histones are randomly and sequentially deposited to assemble “new” nucleosomes; the H3-H4 tetramer and H2A-H2B dimers are deposited on DNA in a chaperone-dependent manner, and this nucleosome assembly is called replication-coupled nucleosome assembly (RCNA) (Figure 2A) [21]. The RCNA process is important for both epigenetic information transmission and genome integrity [22]. In some DNA damage reactions, parental histones must be removed. The removal of histones at DNA damage sites is considered to have many similarities with the RCNA process. In addition, nucleosome assembly during gene transcription and histone exchange can occur throughout the cell cycle and is named replication-independent nucleosome assembly (RINA) (Figure 2B) [21].

## 4. The Role of Histone Modification in Nucleosome Assembly

### 4.1. Influence of Histone Modification on the Deposition of Newly Synthesized Histones

Packaging DNA into the chromatin structure is an important step in DNA replication, which not only ensures the high compaction of DNA but also the correct transmission of epigenetic information to daughter cells [23,24,25,26]. The replication of DNA to the correct packaging of chromatin structure depends on the precise modification, transportation, and assembly of newly synthesized histones. In addition to recycling parental histones, chromatin assembly on replication forks requires the deposition of newly synthesized histones. The expression of canonical histones is activated in the late G1/early S phase to ensure the rapid supply of histones during replication and is inhibited in G1, G2, and early mitosis to prevent the adverse consequences of excess histones on DNA metabolism [27,28].

**Figure 3:**
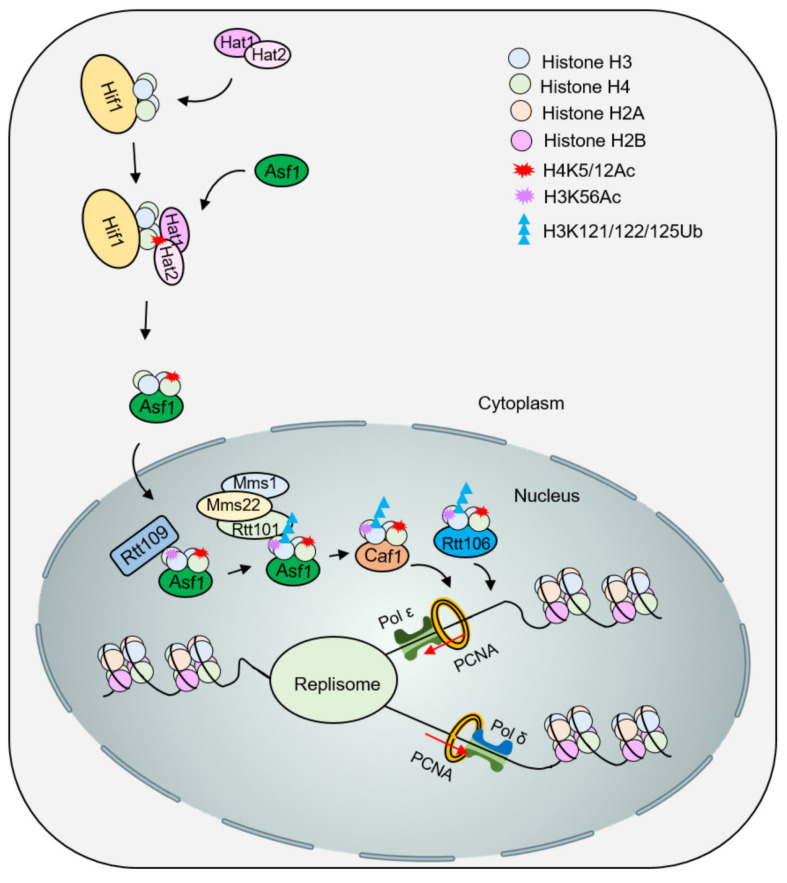
The function of histone modification in nucleosome assembly in yeast. The new H3--H4 is acetylated by the acetyltransferase Hat1/Hat2, and Asf1 promotes nuclear import of H3-H4K5/12ac. Subsequently, acetylation of H3K56 by Rtt109-Vps75 promotes ubiquitination of H3K121/122/125 by Rtt101^Mms1/Mms22^. The modified H3-H4 dissociates from Asf1 and is presented to the chaperones Caf1 and Rtt106 to deposit histones on DNA for nucleosome assembly.

Newly synthesized histones travel with molecular chaperones from the cytoplasm to the nucleus and are modified after translation to facilitate chromatin deposition. In particular, acetylation of the amino terminus tail of H3 and H4 plays an important role in chromatin assembly [29]. The histone acetyltransferase Hat1 can form the Hat1-Hat2 complex with the histone partner Hat2 to acetylate lysine 5 (H4K5) and 12 (H4K12) of histone H4 [30]. In budding yeast, lysine 9 and 27 of histone H3 are acetylated by the acetyltransferases Rtt109 and Gcn5 [31,32], and the acetylation of H4K91 by Hat1 and H3K56 by Rtt109 are both important in replication-coupled chromatin assembly [33,34,35]. H3 can also be acetylated at lysine 14 and 18 in some mammals [36]. In yeast, most newly synthesized histones H3 are acetylated at lysine 56, which is also a marker of newly synthesized histones. However, the acetylation of H3K56 in humans is less than 1.5%, and SETDB1-mediated monomethylation of H3.1K9 marks newly synthesized histones [33]. The histone chaperone Asf1 binds to newly synthesized H3-H4 dimers and presents them to Rtt109 for acetylation [37]. H3K56 acetylation increases the binding affinity of dimer H3-H4 for histone deposition factors CAF-1 and Rtt106, as well as the binding of CAF-1 to chromatin [38]. The Rtt101^Mms1/Mms22^ complex can facilitate this process by preferentially binding and ubiquitinating H3K56ac at lysine 121/122/125, which weakens the interaction of Asf1-H3-H4 and promotes the transfer of H3-H4 to downstream chromatin assembly factors, such as Rtt106 (Figure 3) [39]. Notably, H3K56 acetylation is not essential, in part because GCN5-mediated acetylation of the amino terminus of H3 also increases the binding affinity of the dimer H3-H4 for CAF-1 and Rtt106 [32].

CAF-1 is a highly conserved histone chaperone that plays a role in the deposition of newly synthesized histones through the interaction between its subunits and H3-H4 dimers [40,41,42]. CAF-1 is recruited to the replication fork through direct interaction with PCNA [43,44], and promotes nucleosome assembly through interaction with Asf1. The CAF-1-Asf1 histone deposition complex binds to a single H3-H4 dimer, and CAF-1’s ability to form homodimers may provide the second H3-H4 dimer required for the deposition of (H3-H4)_2_ tetramer [45]. In addition, in vitro interaction analysis has shown that CAF-1 can also bind (H3-H4)_2_ tetramers in monomer form [46]. Interestingly, the binding of H3-H4 to Asf1 stimulated the binding of Asf1 and CAF-1, while the binding of H3-H4 to CAF-1 was mutually exclusive with Asf1, indicating that the H3-H4 transfer process from Asf1 to DNA occurs through CAF-1 [45]. In yeast, the histone chaperone Rtt106 interacts with CAF-1. Rtt106 can also form homodimers, which interact with the K56 region of histone H3 via the double pleckstrin homology domain and bind directly to the newly synthesized (H3-H4)_2_ tetramer. After the acetylation of H3K56, the affinity between Rtt106 and H3-H4 was enhanced [47,48]. The roles of CAF-1 and Rtt106 in new histone deposition are redundant, and only the simultaneous deletion of both complexes will affect histone deposition. Additionally, FACT participates in the deposition of new histones by forming complexes with CAF-1 or Rtt106 and H3K56Ac-H4 (but not Asf1) [22,49].

Replication protein A complex (RPA) can regulate DNA metabolism [50], including three subunits of Rfa1, Rfa2, and Rfa3 in yeast, which bind single-stranded DNA to replication forks and mediate replication movement. The initial replication-coupled nucleosome assembly begins with the deposition of histones H3-H4 onto the replicated DNA, followed by the rapid incorporation of histones H2A-H2B. RPA can directly bind the unmodified H3-H4 histone complex. In vitro experiments have shown that RPA can promote the formation of single-stranded DNA-(H3-H4) complex and can quickly connect to double-stranded DNA. In this process, a series of H3-H4 chaperones are recruited (RPA subunit Rfa2 can bind the three histone chaperones CAF-1, FACT, and Rtt106) to assist in the assembly of new nucleosomes [51]. The above research shows that the main function of RPA is to provide a “platform” for the incorporation of histones into the replication fork through the coupled nucleosome accompanied by DNA replication. It provides a good model for explaining how epigenetic information is assembled and transmitted during chromatin replication.

**Figure 4:**
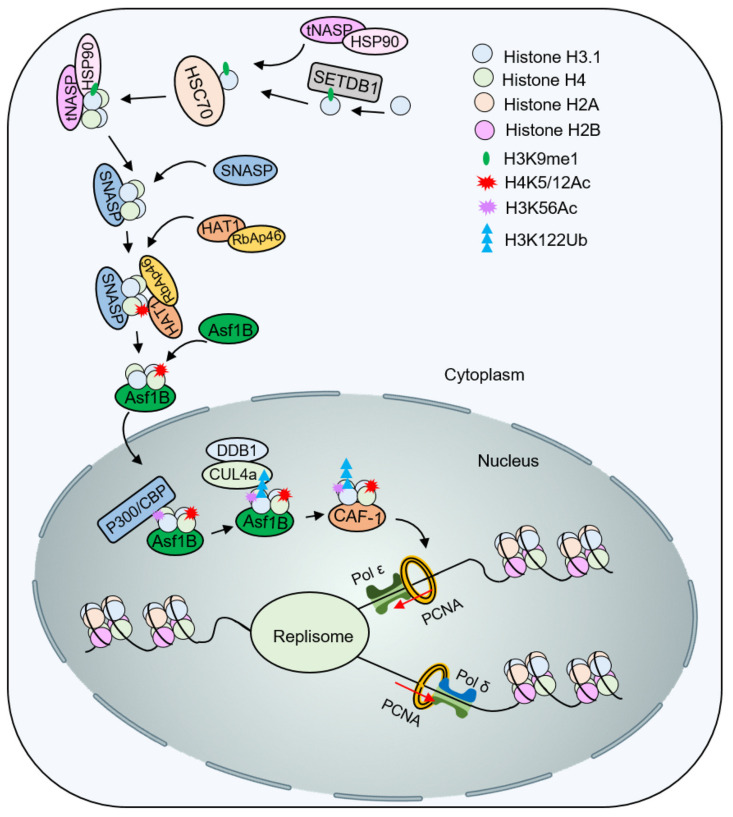
Histone modifications function in nucleosome assembly in mammals. After H4 acetylation on K5 and K12 by HAT1, Asf1 and Importin-4 help nuclear import of H3-H4K5/12ac. Subsequently, acetyltransferase p300/CBP catalyzes the acetylation of H3K56 to promote the ubiquitination of H3K122 by Cul4A^DDB1^. The modified H3-H4 is presented to the histone chaperone CAF-1 to assemble the nucleosome.

In humans, two subtypes of NASP have been identified: sNASP (somatic NASP) and tNASP (testicular NASP) [52]. HSC70 binds to the new histone H3.1, promoting its folding. The newly synthesized histone H3.1 is then presented to HSP90, which, together with the helper chaperone tNASP, promotes the formation of the H3.1-H4 dimer [44]. sNASP is a H3-H4 histone chaperone in cytoplasmic histone processing. sNASP binds the H3.1-H4 heterodimer and presents it to RbAp46 [53]. RbAp46 recruits Hat1 and catalyzes the acetylation of H4K5/12. The histone chaperone Asf1 binds to H3-H4K5/12ac and promotes new histone nuclear import with Importin-4 [54]. In the nucleus, p300/CBP binds and catalyzes acetylation of H3K56, which can further promote Cul4A/DDB1 to catalyze ubiquitination of H3K122 [55]. Acetylation and ubiquitination enable H3.1-H4 to dissociate from Asf1 and be presented to the histone chaperone CAF-1, which is eventually deposited on replicated DNA and involved in the assembly of replication-coupled nucleosomes (Figure 4).

### 4.2. Role of Histone Modification in DNA Damage Repair during Nucleosome Assembly

DNA replication stress poses a threat to the transmission of genetic information [56]. For example, replication stress may contribute to the development of tumors by promoting changes in histone-related epigenetic marker patterns. When the replication fork encounters obstacles, it inevitably enters a stagnant state, which is prone to collapse, leading to DNA damage or genomic instability [57]. Therefore, these obstacles must be repaired or bypassed to restore normal DNA replication [58]. This replication fork damage bypass occurs through different mechanisms, either by break-induced replication using the DNA polymerase Polδ subunit, yeast Pol32 or human POLD3, or by switching to a sister chromatid template to bypass the damage site [59,60,61]. These mechanisms all occur in the context of nucleosome assembly; thus, histone modification plays an important role in DNA damage repair (DDR) and is one of the criteria for selecting damage repair pathways. After DNA damage, the damaged site is marked by histone modification to regulate the signaling pathway in a timely manner and provide support for the assembly of effector proteins [62].

During DNA replication, the MCM2-MCM7 complex is loaded at replication initiation under the regulation of the origin recognition complex (ORC), Cdc6, and Cdt1 to form a prereplication complex (pre-RC). Then, the MCM complex is phosphorylated by DDK and CDK. Cdc45 is recruited to the MCM in a SLD3-dependent manner, and the GINS complex is recruited to the MCM complex by phosphorylated Sld2 and Sld3 together with Dbp11 to assemble the CMG (CDC45-MCM-Gins) complex [63,64]. Double-stranded DNA unwinds into single-stranded DNA (ssDNA), and RPA rapidly binds to the newly formed ssDNA, protecting it from damage and generating secondary structures. Subsequently, DNA polymerase α (Polα) initiates DNA synthesis, DNA polymerase ε (Polε) continuously synthesizes the leading strand, and DNA polymerase δ (Polδ) synthesizes the legging strand [65]. Ctf4, a yeast homolog of human AND1, links CMG helicase to Polα polymerase to form trimers involved in DNA replication [66].

**Figure 5:**
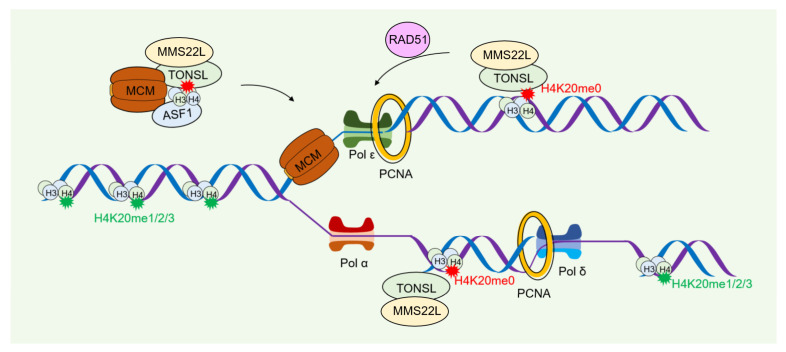
DNA damage repair by chromatin assembly in human. Encounter of a replication fork with DNA lesions that hamper its advance triggers the DNA damage repair (DDR) response. In human cells, MMS22L-TONSL mediates the deposition of newly synthesized histones, binds to unmethylated H3-H4K20 (H3-H4K20me0), and promotes RAD51 recruitment and replication fork recombination repair.

The human MMS22L-TONSL complex is located in the replication fork and increases enrichment when DNA damage occurs [67]. It has a similar function to the Rtt101/Mms1/Mms22 complex in yeast. It has been found that the absence of the MMS22L-TONSL complex affects replication fork stability in the context of CPT stimulation [68]. The proportion of H3K56ac modification in human cells is less than 1.5% of the total H3 [36], and this proportion varies little throughout the cell cycle [69]. However, the unmethylated H3-H4K20 histone is methylated at the late G2/M stage [70]. In particular, MMS22L-TONSL is able to bind not only to newly synthesized histones as part of a predeposited complex with MCM2 and ASF1, but also to H4K20me0 on nascent chromatin. MMS22L and TONSL were necessary for the recruitment and homologous recombination of RAD51 [70,71] (Figure 5).

**Figure 6:**
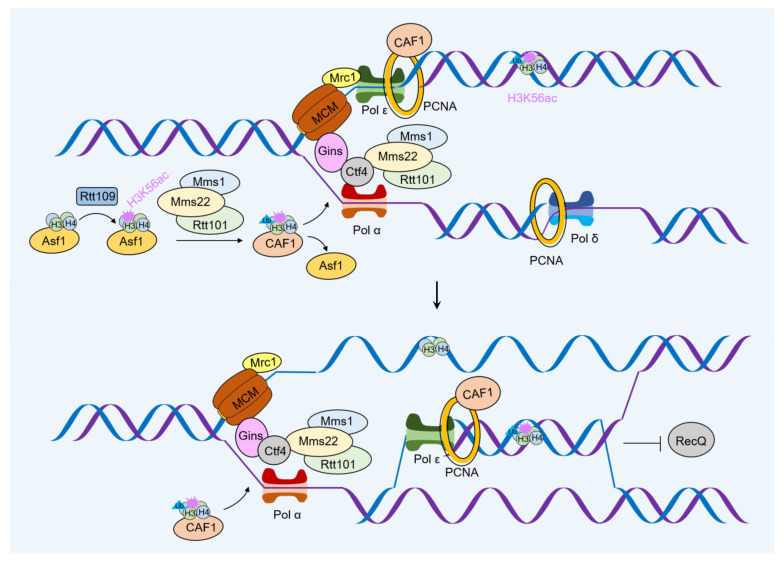
DNA damage tolerance by chromatin assembly in yeast. In yeast, replication-coupled, newly synthesized histones are deposited at the fork to mark chromatin with acetylated H3K56, which in turn activates ubiquitin ligase activity in the Rtt101^Mms1/Mms22^ complex. Ubiquitination of Mrc1 and Ctf4 by unknown factors causes uncoupling of helicase CMG with polymerase and promotes recombination repair of bypass. In addition, the chromatin assembly factor CAF-1 interacts with the D-loop of the RecQ helicase by assembling the nucleosome onto the D-loop and abolishing the D-loop dissociation activity.

Here, we describe the role of a typical histone modification in DDR. H3K56ac is one of the earliest core modifications described in yeast [72] and is deacetylated by the deacetylases Hst3 and Hst4 at the end of S/G2. During DNA damage, Hst3/Hst4 is downregulated in a checkpoint-dependent manner [73], suggesting that H3K56ac modification is crucial in the DNA damage reaction. In fact, yeast cells with defective H3K56 acetylation are highly sensitive to DNA damage agents such as MMS and CPT [74]. Genetic analysis showed that H3K56ac was upstream of the Rtt101/Mms1/Mms22 ubiquitin ligase complex signaling pathway, which is resistant to genotoxic agents [75,76]. Direct evidence for the involvement of H3K56ac in DDR is that fully acetylated H3K56 in vitro increases exposure to DNA sites [77]. This function is unlikely to be related to the role of chromatin assembly in replication fork stability, as cells lacking CAF-1 and Rtt106 are much less sensitive to MMS and CPT than H3K56 acetylated mutants [78]. Members of the Asf1/Rtt109/H3K56ac/Rtt101^Mms1/Mms22^ pathway are required in the process of MMS and CPT-induced DNA damage [79,80]. Deletion mutations in this pathway can disrupt checkpoint recovery after drug therapy, demonstrating that this pathway plays an important role in the mechanism of DDR template conversion. H3K56ac deposition appears to promote the ubiquitination of some unknown substrate to uncouple the replicating helicase with the polymerase as a prerequisite for blocking the recombinant bypass of the lesion (Figure 6). Consistent with this model, the interaction of Rtt101^Mms1/Mms22^ with Ctf4 via the amino terminal tail of Mms22 is necessary for the function of H3K56ac in tolerating replication stress. Ubiquitination of unknown factors is used for Mrc1 and Ctf4 to uncouple the helicase CMG with the polymerase and facilitate recombination repair bypass [81].

Histone chaperone FACT is ubiquitinated by Rtt101 in a manner independent of Mms1/Mms22 [82]. Histones are also potential targets for ubiquitination. Studies have found that human histones are ubiquitinated by Cul4A/DDB1 in UV-induced photodimers, and this modification weakens their interaction with DNA and promotes the recruitment of repair proteins [83,84]. FACT is mainly involved in the deposition of newly synthesized H3-H4. In yeast, Spt16 interacts with Pob3 to form FACT, which is a conserved histone chaperone that plays an important role in DNA regulation [85,86]. Deletion of FACT disrupts the chromatin structure of the gene-coding region [87]. Studies have shown that Spt16 is involved in chromatin remodeling in DDR through ubiquitination of H2B [88,89,90]. At the same time, FACT can regulate DDR mediated by homologous recombination (HR) and base excision repair (BER), which proves that FACT is essential for damage repair [91]. Asf1 is critical for histone modification, histone deposition, and DNA replication. Its function in heterochromatin silencing was first identified in yeast [92] and later found as a replication-coupled assembly factor (RCAF) in Drosophila [93]. Asf1 can bind to the histone H3-H4 dimer with Mcm2-7. Histone H3-H4 was modified with specific parental labeling (H4K16ac and H3K9me3) under hydroxyurea treatment, leading to the accumulation of replication forks [94,95]. It is suggested that Mcm2-H3-H4-Asf1 is an intermediate in parental histone assembly and may promote DNA unwinding through its ability to transfer histones during chromatin assembly. CAF-1 promotes a Rad51-dependent replication fork bypass repair pathway [96]. The interaction between CAF-1 and the RecQ helicase Bloom in human cells is conserved, and both factors accumulate in the DNA replication center through replication stress and promote cell survival [97].

## 5. Histone Modification and Cancer

Histone modification is involved in chromatin remodeling [98], thereby altering chromatin status and gene expression [99] and is very important for gene regulation and genomic stability. Abnormal histone modification can cause abnormal chromatin status or genomic instability, which is often believed to be closely related to the occurrence and development of cancer [100,101,102].

### 5.1. Histone Methylation in Cancer

Histone methylation, including monomethylation, demethylation, and trimethylation, is regulated by methyltransferases and demethylases and occurs mainly on lysine residues of H3 and H4 [103]. H3K4me1/2/3, H3K36me1/2/3, and H3K79me1/2/3 are transcriptionally active marks, while H3K9me1/2/3 and H3K27me3 are transcriptionally repressive marks [9]. Histone methylation disorder leads to the destruction of gene expression and genomic stability, and the abnormal modification of histone methylation in tumor cells can alter cancer development (Table 1). For example, decreased H3K27me3 and increased H3K4me3 activate the Wnt/β-catenin signaling pathway to promote colorectal cancer cell development [104]. Mutations in histone methylation sites (H3K27M, H3K27I, etc.) are present in approximately 30% of children with glioblastoma [105]. NSD2 maintains genome integrity and reduces disease incidence by methylating H3K36 and DOT1-mediated H3K79 methylation in response to UV radiation-induced DDR [106]. Under normal physiological conditions, the number of H3K9me3 increases dramatically over time at the site of DNA double-strand break damage and participates in DDR. In contrast, in the environment of abnormal tumor cell metabolism, abnormal H3K9me3 inhibits DNA repair [107].

Histone methylation has been used as a promising target for cancer therapy. A large number of methyltransferase inhibitors have been developed and entered clinical trials, mainly against H3K27 and H3K79 methyltransferase, and arginine methyltransferase [108]. EZH2, a methyltransferase of H3K27, is involved in tumor occurrence, metabolism, drug resistance, and immune regulation [109]. Therefore, targeting EZH2 for cancer therapy has become a hot research topic. Strategies for EZH2 inhibitors include targeting methyltransferase activity (GSK126, GSK343, EPZ011989, et al.), breaking PRC2’s structure (SAH-EZH2, Astemizole, MAK683, et al.), or triggering EZH2 degradation (GNA022, ANCR, FBW7, et al.) [109]. For example, EZH2 inhibitor GSK343 can decrease self-renewal and increase sensitivity to chemotherapy in colorectal cancer cells [110]. Some studies have also shown that EZH2 has an antitumor effect [111,112]. For example, GSK126 can increase the number of myeloid-derived suppressor cells (MDSC) and decrease the number of IFNγ^+^CD8^+^ T cells, leading to the failure of antitumor therapy. Interestingly, when combined with neutralizing antibodies against the myeloid differentiation antigen GR-1, MDSC-mediated immunosuppression was mitigated and increased the therapeutic effect of GSK126 [113]. Developing a multi-drug combination therapy strategy may address the limitations of single drug therapy. These studies indicate that histone methylation modification plays an important role in the development and prevention of cancer.

**Table 1:** The role of genes encoding histone methyltransferase in human cancer.

Gene	Tumor	Role	Reference
*SETD1A*	Colorectal cancer, Lung cancer, Gastric cancer	Oncogenic	[114,115,116]
*SETD1B*	Pancancer	Suppressor	[117]
*MLL1*	Breast cancer, Cervical carcinoma, Acute myeloid leukemia	Oncogenic	[118,119,120]
*MLL2*	Bland cancer, Prostate cancer	Suppressor	[121,122]
*MLL3*	Nasopharyngeal carcinoma, Breast cancer, Pancreatic cancer, Colorectal cancer	Ambiguous	[123,124,125,126]
*MLL4*	Lung cancer, Medulloblastoma,	Suppressor	[127,128]
*SMYD2*	Lung cancer, Cervical cancer	Oncogene	[129,130]
*SET7*	Glioma, Colorectal cancer, Lung cancer	Suppressor	[131,132,133]
*SET9*	Glioma, Lung cancer, Breast cancer	Suppressor	[131,133,134]
*SMYD3*	Pancreatic cancer, Lung cancer, Breast cancer	Overexpression	[135,136,137]
*SUV39H1*	Cervical cancer, Prostate cancer, Melanoma	Oncogene	[138,139,140]
*SUV39H2*	Colorectal cancer, Osteosarcoma, Lung cancer	Oncogene	[141,142,143]
*G9A*	Colorectal cancer, Bladder cancer, Lung cancer, Breast cancer	Oncogene	[144,145,146,147]
*SETDB1*	Lung cancer, Gastric cancer, Colorectal cancer	Oncogenic	[148,149,150]
*PRDM3*	Pancreatic ductal adenocarcinoma	Suppressor	[151]
*PRDM16*	Kidney cancer, Prostrate cancer, Lung cancer	Suppressor	[152,153,154]
*EZH1*	Breast cancer, Hepatocellular carcinoma	Oncogenic	[155,156]
*EZH2*	Lung cancer, Hepatocellular carcinoma, Breast cancer, Gastric cancer, Colorectal cancer	Oncogene	[156,157,158,159,160]
*SETD2*	Prostate cancer, Pancreatic cancer, Leukemogenesis, Hepatocellular carcinoma	Suppressor	[161,162,163,164]
*NSD1*	Pancreatic cancer, Laryngeal cancer	Oncogenic	[165,166]
*NSD2*	Lung cancer, Colorectal cancer, Breast cancer, Renal cancer, Osteosarcoma, Prostate cancer	Oncogene	[167,168,169,170,171,172]
*NSD3*	Lung cancer, Breast cancer, Colorectal cancer, Pancreatic cancer	Oncogene	[173,174,175]
*SETD3*	Breast cancer, Hepatocellular carcinoma	Overexpression	[176,177]
*ASH1L*	Prostate cancer, Leukemia	Oncogenic	[178,179]
*SETMAR*	Acute myeloid leukemia	Suppressor	[180]
*PRDM9*	Pancancer	Overexpression	[181]
*DOT1L*	Prostate cancer, Colorectal cancer, Gastric cancer, Ovarian cancer, Breast cancer, Acute myeloid leukemia	Overexpression	[119,182,183,184,185,186]
*SET8*	Prostate cancer, Hepatocellular carcinoma, Breast cancer	Oncogenic	[187,188,189]
*SUV4-20H2*	Hepatocellular carcinoma, Breast cancer	Suppressor	[190,191]

### 5.2. Histone Acetylation in Cancer

Acetylation is one of the main modifications of histones and is strictly regulated by histone acetyltransferases (HAT) and histone deacetylases (HDAC) to maintain the normal acetylation state, thus controlling the initiation and shutdown of gene transcription. HATs transfer the acetyl group from acetyl-CoA to the amino terminal of the specific lysine residue of the histone, generating an acetate bond. Acetylation is a key epigenetic mechanism in gene regulation [192] and regulates chromatin structure and function through transcriptional capacity [193,194]. Abnormal histone acetylation can disrupt cell homeostasis and affect cell metabolism and gene regulation [195]. Cumulative evidence suggests that abnormal expression of histone modification enzymes is closely related to tumor development (Table 2). The current antitumor treatment of histone acetylation as a therapeutic target is expected to be achieved through the development of HAT and HDAC inhibitors. The first HDAC inhibitor approved for clinical treatment was suberoylanilide hydroxamic acid (SAHA), and more drugs are being developed, such as YF479, which has good antitumor activity and can inhibit the recurrence and metastasis of breast cancer [196,197]. Thus, histone acetylation modification plays a significant role in the occurrence and development of cancer.

### 5.3. Histone Ubiquitination in Cancer

Ubiquitin (Ub) exists widely in eukaryotes, and ubiquitination is also one of the main posttranscriptional modifications. Posttranslational modification of proteins is a reversible, dynamic process. Histone ubiquitination is dynamically regulated by ubiquitination enzymes and deubiquitination enzymes and can participate in most intracellular processes, including protein degradation, intracellular signaling, endocytosis, and DNA damage reactions [216,217,218]. Histone ubiquitination is the core event of DDR, and DNA damage requires a large number of ubiquitin molecules, which are crucial for preventing abnormal DNA repair and maintaining genomic stability [219]. Histone H3 ubiquitination enzymes mainly include NEDD4 and CUL4A. NEDD4 ubiquitinates histone H3 on lysine 23/36/37 residues in a glucose-dependent manner, specifically recruiting the histone acetyltransferase GCN5 for subsequent H3 acetylation. This mechanism can regulate gene transcription and tumorigenesis in cancer [220]. The RNA demethylase ALKBH5 and the USP22/RNF40 axis regulate histone H2AK119 monoubiquitination to regulate the expression of key genes involved in DNA repair, thus playing a crucial role in the development of osteosarcoma [221]. Rad6 and Bre1 form a well-characterized H2B monoubiquitin enzyme to degrade histones in DDR reactions [222]. USP11 can deubiquitinate H2AK119 and H2BK120 to separate ubiquitin molecules from histones and maintain genomic stability [223]. It is worth noting that the existing studies on histone ubiquitination mainly focus on histone H2A/H2B, and the discovery of histone H3 ubiquitination and the study of its mechanism are also gradually deepening. However, the regulation of histone H3 deubiquitination remains unclear.

### 5.4. Histone Phosphorylation in Cancer

Histone phosphorylation occurs on serine and tyrosine residues of histones and has been shown to be involved in many cellular life activities, including DNA damage repair, gene transcription, chromatin maintenance and aging, through histone methylation [224,225]. For example, PRK-mediated H3T11 phosphorylation (H3T11ph) hastens the removal of repressive histone H3 lysine 9 (H3K9) methylation by JMJD2C, demonstrating a unique mechanism by which histone phosphorylation activates gene expression. Importantly, the level of H3T11ph correlates with prostate cancer malignancy, suggesting that inhibition of H3T11ph may be a promising therapeutic target [226]. Phosphorylated H3.3 (H3.3S31ph) enhances the binding of the methyltransferase SETD2 to histone proteins, thus promoting gene transcription and highlighting the causal role of H3.3 phosphorylation in tumor metastasis [227]. H3.3S31ph is also involved in the regulation of heterochromatin regions and reduces the demethylation of H3K9me3 to maintain chromatin integrity by downregulating the activity of KDM4B [228]. Pyk1-catalyzed H3T11ph can weaken the binding of Dot1 to chromatin and reduce Dot1-mediated H3K79me3, leading to suppression of autophagy-related gene transcription and uncovering histone modification crosstalk in response to cell metabolism [229]. Additionally, a recent study showed that phosphorylation of histone H3 at serine 10 inhibits methylation of histone H3 at adjacent arginine 8, providing a framework for understanding the effects of phosphoserine on the methylation of adjacent amino acid residues and arginine [230]. In order to function, histone phosphorylation may antagonize its methylation.

## 6. Conclusions and Perspectives

Nucleosome assembly is a complex and highly regulated process in eukaryotes. Nucleosome assembly requires precise regulation by histone-modifying enzymes, histone chaperones, and histone modifications. In recent years, yeast-based experiments have provided new insights into how to regulate the assembly of novel H3-H4 through histone modification and molecular chaperones. Additionally, nucleosome reassembly maintains replication fork stability, but the mechanism remains elusive. Similarly, histone epigenetic modifications affect the complexity and correlation of newly assembled chromatin structures during DDR [231]. In fact, some aspects of the replication-dependent chromatin assembly process are not discussed here because there is still no evidence that they are associated with the progression and stability of replication forks. Finally, it is worth mentioning that although we focus on how chromatin assembly regulates DNA replication, the effects are mutual. For example, replication stress resulting from replication disorders promotes heterochromatin formation. The combination of genetic and biochemical approaches with genome-wide analysis may help reveal the dynamics of chromosomal remodeling in these different scenarios and understand the molecular mechanism of how defects in replication-coupled chromatin assembly lead to genetic diseases, cancer, and aging.

Epigenetic modification of histones is closely related to disease pathogenesis and can be a molecular signature in cancer [232]. The diversity of histone modifications provides new molecular targets for the treatment of various diseases [233]. It is worth noting that many drugs targeting histone modifications have been developed and used in clinical research in the past decade, which is sufficient to show that histone modification plays a very important role in disease treatment. Therefore, understanding the basic mechanisms controlling epigenetic modification changes will bring new breakthroughs and advances in drug development and treatment of cancer and other human diseases [234]. The development of epigenetic drugs creates a new avenue for the treatment of diseases, which is a huge leap forward in the extension of basic scientific research to clinical drug development. Meanwhile, numerous studies have found that histone modification tandem is closely related to the development and pathogenesis of various diseases, indicating a new direction for the research and development of histone modification inhibitors.

## Figures and Tables

**Table 2:** The role of genes encoding histone acetyltransferase in human cancer.

Gene	Tumor	Role	Reference
*HAT1*	Liver cancer, Pancreatic cancer, Cholangiocarcinoma	Overexpression	[198]
*GCN5*	Colorectal cancer	Overexpression	[199]
*PCAF*	Lung cancer, Esophageal cancer	Ambiguous	[200,201]
*Tip60*	Prostate cancer, Breast cancer	Ambiguous	[202,203]
*MOF*	Bladder cancer, Endometrial cancer, Renal cell carcinoma	Suppressor	[204,205,206]
*MOZ*	Gastrointestinal stromal tumor, Acute myeloid leukemia	Oncogene	[207,208]
*MORF*	lymphoma	Oncogene	[209]
*HBO1*	Lung cancer, Osteosarcoma, Hepatocellular carcinoma, Breast cancer	Oncogenic	[210,211,212,213]
*p300*	Esophageal cancer, Prostate cancer, Lung cancer	Oncogenic	[201,214,215]
*CBP*	Colorectal cancer	Oncogenic	[183]

## Data Availability

Not applicable.
